# Supplementary material for: Knowledge, attitudes, and positions of religious leaders towards female genital cutting: A cross-sectional study from the Kurdistan Region of Iraq
Source: PLoS One. 2022 Nov 1;17(11):e0265799. doi: 10.1371/journal.pone.0265799 (PMC9624424; doi:10.1371/journal.pone.0265799)
Supplement: S1 File — (DOCX) [file pone.0265799.s001.docx]

رووثيَوى زانيارى و بووضوون و رِاى ماموستايانى ئايينى سةبارةت بة خةتةنة كردنى ميَينة

زانيارى كةسى

تةمةن:

خةلكى ناوضةى:

ئاستى خويَندةوارى:

شويَنى كاركردن (شوين و ناوى مزطةوت):

شويَنى دانيشتن:

بةرثرسيارةتى:

سالانى خزمةت:

سوودةكان و زةرةكانى خةتةنةكردنى ميَينة:

سوودةكان بريتين لة: (تكاية دةتوانى زياتر لة يةك هةلَبذاردة هةلَبذرى كة دةزانى راستة)

☐كةمكردن يان ريَكخستنى مةيلى زيادى جنسى ئافرةتان بوَ ئةوةى لة طوناة و كيَشة كوَمةلايةتييةكان دوور بن

☐بوَ ثاك و خاويَنى زيادتر و نةهيَشتنى بوَنى ناخوَش

☐حةلال بوونى خواردن لة دةستى ئافرةتان

☐بوَ رِاطرتنى ثةردةى كضينى تاكوو هاوسةرطيرى

☐بوَ ريَطرتن لة ثةيوةندى سيكسى لة دةرةوةى هاوسةريةتى

☐بوَ ريَطةطرتن لة ئازارى ثياو (لة بةر ميتكةى طةورة) لة كاتى سيَكس

☐ هى تر، تكاية بنووسة: ............................................................................

☐نازانم

زةرةكانى بريتين لة: (تكاية دةتوانى زياتر لة يةك هةلَبذاردة هةلَبذرى كة دةزانى راستة)

☐هيض زيانيَكى نية بة هيض شيَوةيةك

☐كةمكردن يان لة دةستدانى مةيلى سيَكسى

☐خويَنبةربوون

☐ذان

☐هةوكردن

☐كيَشة دةرونيةكان

☐كيَشةى مندالَبوون لة داهاتوو

☐ هى تر، تكاية بنووسة: ............................................................................

☐نازانم

هوَيةكانى خةتةنةكردنى ميَينة

خةتةنةكردنى ميَينة لة لايةن خةلَك ئةنجام دةدريَت لة بةر :(تكاية دةتوانى زياتر لة يةك هةلَبذاردة هةلَبذرى كة دةزانى راستة)

☐ئةمة ثيَويستيةكى دينية

☐لة كةلتورةوة هاتووة

☐ئةمة شتيَكى ضاوليَكةرية و لة كوَنةوة هةية و كة لة طةلَ دين تيَكةل كراوة

☐بوَ ثاك و خاويَنى و نةهيَشتنى بوَنى ناخوَش

☐كةمكردن و ريَكخستنى هةستى سيَكسى ئافرةت لة بةر ئةوة كة هةستيان بة سروشت زيادترة لة ثياو

☐نازانم

ئةنجامدةرانى خةتةنةى ميَينة

لة كوردستانى عيَراق، خةتةنةكردنى ميَينة لة لايةن ئةمانة ئةنجام دةدريَت: (تكاية دةتوانى زياتر لة يةك هةلَبذاردة هةلَبذرى كة دةزانى راستة)

☐مامانةكان ناو طةرةطةكان ☐ثزيشك و ثةرستارةكان ☐ خةتةنة كةرةكانى ناو خةلك ☐ ثيرةذنةكان

هى تر: ( تكاية بنووسة): ................................................. ☐نازانم

كىَ برَيارى ئةنجامدانى خةتةنةكردن دةدات بوَ كض؟

☐دايك ☐باوك ☐هةردووكيان ☐ داثير ☐ ماموستاى ئاييني ☐هى تر:..................... ☐نازانم

ثةيوةندى خةتةنةكردنى ميَينة بة كوَمةلطا

ئايا خةلك ثرسيار لة سةر ئةم بابةتة ثرسيارتان لىَ دةكةن؟ ☐ بةلىَ ☐ نةخيَر

ئةطةر بةلىَ ضةند: ☐ زوَر بة كةمى ☐هةنديَك جار ☐ زوَرجار

ض كةسيَك و لة ض ئاستيَك ثرسيارتان لىَ دةكات؟ (تكاية دةتوانى زياتر لة يةك هةلَبذاردة هةلَبذرى كة دةزانى راستة)

☐ثياو ☐ئافرةت ☐ هةذار ☐زةنطين ☐ خويَندةوار ☐ نةخويَندةوار ☐ شارنشين ☐ طوندنشين

لة ض سةردةميَك ثرسيار كردن زيادتر بووة؟ ☐ ئيَستا ☐ رابردوو

ئايا هيض كةسيَك طلةيي لة نةريتة كردووة لاى ئيَوة؟ ☐ بةلىَ ☐ نةخيَر

ئةطةر بةلىَ طلةييةكة ضى بووة ؟

☐ ئافرةتان لة كةم بوونى هةستى سيَكسى خوَيان ☐ ثياوان لة كةم بوونى هةتى سيَكسى هاوسةرةكانيان

☐ هى تر: (تكاية بنووسة)...............................................

ئةطةر نةخيَر ثيَت واية بوَ؟

☐ ضونكة خةتةنةكردنى ئافرةتان هيض كيَشيةكى نية

☐ خةلَك باسى ئةم كيَشانة ناكان ضونكة بابةتيَكى هةستيارة و شةرم دةكةن

ئايا خةلك طلةيي لة ئةنجام نةدانى خةتةنةكردن بوَ ئافرةتان دةكةن؟ ☐ بةلىَ ☐ نةخيَر

ئةطةر بةلىَ ض كيَشةيةك؟..............................................................................................

بةربةلاوى خةتةنةكردنى ميَينة لة هةريَمى كوردستانى عيَراق

ئايا خةتةنةكردنى ميَينة لة كوردستان بة ربةلاوة؟ ☐ بةلىَ ☐ نةخيَر ☐ نازانم

ئايا خواست لة سةر خةتةنة كردنى ميَينة زوَرة يان كةمة؟ ☐ بةرةو زوَر بوونة ☐ بةرةو كةم بوونةوة

بةربةلاوى خةتةنةكردنى ميَينة

لة هةوليَر ☐ بةربةلاوة ☐ بةربةلاو نية

لة دهوَك ☐ بةربةلاوة ☐ بةربةلاو نية

لة سليمانى ☐ بةربةلاوة ☐ بةربةلاو نية

لة كةركوك ☐ بةربةلاوة ☐ بةربةلاو نية

لة شارةكان ☐ بةربةلاوة ☐ بةربةلاو نية

لة طوَندةكان ☐ بةربةلاوة ☐ بةربةلاو نية

هوَيةكانى جياوازى لة بةربةلاوى خةتةنةكردنى ميَينة لة شويَنة جياجياكانى هةريَمى كوردستان ضين: (تكاية دةتوانى زياتر لة يةك هةلَبذاردة هةلَبذرى كة دةزانى راستة)

☐جياوازى لة مةزهةب

☐جياوازى لة ئيلتيزام بة مةزهةبى شافعى

☐جياوازى لة ئاستى ئيمان

☐جياوازى لة ئاستى خويَندةوارى و وشيارى خةلَك

☐جياوازى لة كاريطةرى كةمثينةكانى ئاطاداركردنةوة دذى خةتةنةكردنى ميَينة

☐جياوازى لة كةش و هةوا لة شويَنة جياجياكانى كوردستان

☐جياوازى لة داب و نةريت

☐نازانم

رِاى تايبةتى خوَت سةبارةت خةتةنةكردنى ميَينة

رِاى تايبةتى خوَت سةبارةت بة خةتةنةكردنى ميَينة ضية؟

☐ثشتطيرى دةكةم ☐ دذى مة ☐ريَطة ثيَدراوة و بة ثىَ ئارةزووى كةسةكةية ☐ هيض رِايةكم نية ☐ هى تر:......................................................

ئةطةر ثشتطيرى دةكةى هوَى ضية؟ ☐ ئايين ☐ كةلتوور

ئةطةر دذى هوَى ضية؟

☐ ناروونى لة بةلَطةى دينى لة سةر ئةم بابةتة

☐ئةوة تةنها نةريتيَكى كةلتورية و كاريطةرى خراثى هةية

ئةطةر ريَطة ثيَدراوة و بة ثىَ ئارةزووى كةسةكة هوَى ضية:

☐ ئةو حةديسانةى كة هانى ئةم كارة دةدةن لاوازن

☐ هيض حةديسةك ريَطرى نةكردووة

ئةطةر هيض رايةكت نية هوَى ضية: ☐زور زانيارييم لة سةر نية ☐هة تر:............................................

قةدةغة كردنى خةتةنة كردنى ميَينة بة ياسا

ئايا ثيَت باشة ياسايةك هةبيت خةتةنةكردنى ميَينة قةدةغة بكات؟ ☐ بةلىَ ☐ نةخيَر

ئةطةر نة بوَ؟ (تكاية دةتوانى زياتر لة يةك هةلَبذاردة هةلَبذرى كة دةزانى راستة)

☐ياسايةكة دذى شةريعةت و ريَنمايي ئايينى دةبيت

☐ياساكة كارى ثيَ ناكريَت

☐ياساكة طوَمان دروست دةكات و خولك دذى دةوةستن

☐هى تر:................................................................................

ثيَويستى كضان و ئافرةتان بة خةتةنة كردن

ئةطةر خةتةنة كردن دةبيَت ئةنجام بدريَت ض كةسيَك ثيَويستى بة خةتةنة كردنة؟

☐هةموو كضان و ئافرةتان

☐هيضكاميان

☐ئةوانةى ميتكةى طةورةيان هةية و زوَر هةستيارة و ئيزعاجى بوَ ثياو لة كات سيَكس دروست دةكات

☐شارةزايانى تةندروستى (ثزيشك يان ثةرستار) دةبيَت برِيار بدةن كىَ ثيَويستى ثيَية

☐ئةوانةى لة شويَنة طةرمةكان دةذين ضونكة كض زووتر بالغ دةبيت و ئارةزووى سيَكسى زوَرة

☐تةنيا ئةو ذنانةى ئارةزووى سيَكسيان زوَرة و ئةطةرى ئةنجامدانى طوناهيان هةية

☐نازانم

ض كةسيَك دةبيَت خةتةنة كة ئةنجام بدات؟

☐مامان

☐ثزيشك يان ثةرستار

☐ئةو كةسانةى هةر لة كوَن كاريان خةتةنة كردنة

☐ثيرةذنةكان

خةتةنةكردنى ميَينة و ئايينى ئيسلام

بة رِاى جةنابت بووضوونى ئيسلام سةبارةت بة خةتةنةكردنى ميَينة ضية؟

☐واجبة ☐ سونةتة ☐ مباحة ☐ مةكرةمةية ☐ مكروهة

بووضوونى مةزهةبةكان لة سةر ئةم بابةتة ضية؟

شافعى ☐واجبة ☐ سونةتة ☐ مباحة ☐ مةكرةمةية ☐ مكروهة ☐ نازانم

حنفى ☐واجبة ☐ سونةتة ☐ مباحة ☐ مةكرةمةية ☐ مكروهة ☐ نازانم

مالكى ☐واجبة ☐ سونةتة ☐ مباحة ☐ مةكرةمةية ☐ مكروهة ☐ نازانم

حنبلى ☐واجبة ☐ سونةتة ☐ مباحة ☐ مةكرةمةية ☐ مكروهة ☐ نازانم

بةلَطة كانى ئايينى سةبارةت بة خةتةنةكردنى ميَينة

☐حةديسةكان لاوازن

☐حةديسةكان بة روونى باسى خةتةنةكردن بوَ كض يان كوَر ناكات

☐حةديسى كة بة روونى و بة هيَزى هان بدات بوَ خةتةنةكردنى ميَينة نية

☐هيض حةديسةك قةدةغةى نةكردووة

☐لة حةديسةكان نة هان دراية نة قةدةغة كراية

☐ئامانجى حةديسةكان لة سةرةتاى ئيسلام وابوو كة ئةنجامدانى خةتةنةكردنى ميَينة سنوردار بكريَت و لة بةر ئةوة نةبووة هانى ئةم كارة بدريَت و واجبى بكات

☐هةبوونى حةديس لة سةرى نيشانةى ئةوةية ثةيغةمبةر دروودى خواى لة سةر بيَت دةزانى ئةم كارة ئةنجام دةدريَت كةواتة ريَطة ثيَدراوة

☐ئيسلام تةنيا خةتةنةكردنى سووك و كةمى ثىَ قبوولة

دروستى و ثةيوةندى حةديسةكان لة سةر خةتةنةكردنى ميَينة

1. ئةو حةديسةى باسى ثنج فيترة دةكات ☐ سةحيحة ☐ حةسةنة ☐ زةعيفة ☐تايبةتة تةنيا بة كوران ☐تايبةتة بة هةر دوو كض و كور

2- حةديسى ثةيغةمبةر سةبارةت ئةو ئافرةتةية كة لة مةدينة كارى خةتةنةى دةكرد ☐ سةحيحة ☐حةسةنة ☐ زةعيفة

3- ئةو حةديسةى كة خةتةنةكردنى ميَينة بة مةكرةمة دةزانيَت ☐ سةحيحة ☐حةسةنة ☐ زةعيفة

4- حةديسى لة كاتى طةيشتنى دوو شوينى خةتةنة كراو دةبيَت غوسل بكريَت ☐ سةحيحة ☐حةسةنة ☐ زةعيفة

روَلى ماموستايانى ئايينى لة قةدةغة كردنى خةتةنةكردنى ميَينة (تكاية دةتوانى زياتر لة يةك هةلَبذاردة هةلَبذرى كة دةزانى راستة)

☐ نابيَت قةدةغة بكريَت

☐ماموستايانى ئايينى دةتوانن روَلى كاريطةريان هةبيَت سةبارةت ئةم بابةتة لة بةر ئةم جيَطا تايبةتةى كة لة ناو كوَمةلَطا هةيانة

☐ئيمام و خةتيبةكان دةبيَت روَليان هةبيَت لة قةدةغة كردنى لة وشياركردن و تيَطةياندنى خةلَك كة ئةمة كاريَكى هةلةية و ئايين ئيجبارى نةكردووة

☐ئيمام و خةتيبةكان دةبيَت خةلك هان بدةن بوَ ئةم كارة

☐ماموستايانى ئايينى دةبيَت بطةنة وةلاميَكى يةكلايكةرةوة بوَ ئةوةى ريَنمايي خةلَك بكةن

☐ئيمام و خةتيبةكةكان ناتوانن هيض روَليَك ببينن لة بةر ئةوةى ئةمة شةرعة و ناكريَت دةستكارى بكريَت

☐ئيمام و خةتيبةكان ناتوانن روَليان هةبيت لة بةر ئةوةى ئةم بابةتة زوَر هةستيارة و بابةتى ئايبةت بة ئافرةتانة و لة ناو خةلَك باس ناكريَت

☐ثيَويستة ريَنماييةكى ستاندارد هةبيت سةبارةت بة خةتةنةكردنى ميَينة ثاش ئةنجامدانى تويَذينةوة و خويَندنةوةيةكى باش لة لايةن هةردوو ماموستايانى ئايينى و ثسثورانى بوارى ثزيشكى

☐ هى تر تكاية بنووسة ........................................................................................................

لة كوَتايى زوَر سوثاسى بةريَزتان دةكةين بوَ بةشدارى لةم رِاثرسية. ئةطةر قسةى زيادتر يان تيَبينى وة يان ثشنيارةكت هةية تكاية بنووسة: ............................................................................................................................

......................................................................................................................................................................................................................................................................................................................................................................................................................................................................................................................................................................................................................................................................................................................................................................................................................................................................................................................................................................
